# Fatal toxoplasmosis in *Coendou spinosus* from Southern Brazil: clinical, pathological, and genotype findings

**DOI:** 10.1590/S1984-29612025051

**Published:** 2025-10-13

**Authors:** Renata Fagundes-Moreira, Karina Oberrather, Luan Henker, Luiza Presser Ehlers, Fagner D’ambroso Fernandes, Diego Ferreira Cardoso, Alisson da Rosa Boyink, Fernanda Silveira Flores Vogel, Saulo Petinatti Pavarini, Luciana Sonne, João Fabio Soares

**Affiliations:** 1 Università di Pavia – UNIPV, Dipartimento di Sanità Pubblica, Medicina Sperimentale e Forense, Pavia, Italia; 2 Università Degli Studi di Bari Aldo Moro – UNIBA, Dipartimento di Medicina Veterinaria, Valenzano, Bari, Italia; 3 Universidade Federal do Rio Grande do Sul – UFRGS, Faculdade de Veterinária, Laboratório de Protozoologia e Rickettsioses Vetoriais – Protozoovet, Porto Alegre, RS, Brasil; 4 Auburn University, College of Veterinary Medicine – ACOVM, Anatomic Pathology Resident, Auburn, USA; 5 Universidade Federal do Rio Grande do Sul – UFRGS, Faculdade de Veterinária, Setor de Patologia Veterinária, Porto Alegre, RS, Brasil; 6 Universidade Federal de Santa Maria – UFSM, Departamento de Medicina Veterinária Preventiva, Centro de Ciências Rurais, Laboratório de Doenças Parasitárias – LADOPAR, Santa Maria, RS, Brasil; 7 Zoológico Municipal de Cachoeira do Sul, Cachoeira do Sul, RS, Brasil

**Keywords:** Toxoplasmosis, zoonotic pathogens, wildlife surveillance, Public Health, Coendou spinosus, Toxoplasmose, patógenos zoonóticos, monitoramento da vida selvagem, Saúde Pública, Coendou spinosus

## Abstract

Although toxoplasmosis is well documented in New World non-human primates, data on its clinical impact and genotypic diversity in neotropical rodents remain limited. This study investigated fatal toxoplasmosis in *Coendou spinosus* through histopathological, immunohistochemical, and molecular analyses, with genotypic characterization of the infecting strain. Between September 2016 and October 2019, eight individuals were analyzed, including rescued animals and *post-mortem* cases from a local zoo in Southern Brazil. Tissue samples underwent histopathology and immunohistochemistry using anti-*T. gondii* antibodies, and molecular assays were conducted by nested PCR and PCR-Restriction Fragment Length Polymorphism (PCR-RFLP). Severe necrotizing lesions, especially in the central nervous system, were observed in five animals, associated with abundant bradyzoites and tachyzoites. Molecular detection confirmed *T. gondii* DNA in all animals, and subsequent genotyping revealed a previously undescribed atypical strain in the animal CS8. These findings demonstrate the high mortality associated with *T. gondii* infections, presenting documented clinical signs of toxoplasmosis in *C. spinosus* and highlighting its vulnerability to zoonotic pathogens, particularly due to habitat encroachment and increased interactions with humans and other wildlife.

## Resumo

Embora a toxoplasmose seja bem documentada em primatas não-humanos do Novo Mundo, os dados sobre seu impacto clínico e diversidade genotípica em roedores neotropicais ainda são limitados. Este estudo investigou casos fatais de toxoplasmose em *Coendou spinosus* por meio de análises histopatológicas, imuno-histoquímicas e moleculares, com caracterização genotípica do isolado envolvido. Entre setembro de 2016 e outubro de 2019, oito indivíduos foram analisados, incluindo animais resgatados e casos pós-morte provenientes de um zoológico no sul do Brasil. Amostras de tecidos foram submetidas à histopatologia e à imuno-histoquímica utilizando anticorpos anti-*T. gondii*, e os testes moleculares foram realizados por *nested*-PCR e PCR–Polimorfismo de Comprimento de Fragmentos de Restrição (PCR-RFLP). Lesões necróticas severas, especialmente no sistema nervoso central, foram observadas em cinco animais, associadas à presença abundante de bradizoítos e taquizoítos. A detecção molecular confirmou DNA de *T. gondii* em todos os animais, e a genotipagem subsequente revelou um isolado atípico previamente não descrito no animal CS8. Esses achados demonstram a alta mortalidade associada à infecção por *T. gondii*, apresentando sinais clínicos documentados de toxoplasmose em *C. spinosus* e destacando sua vulnerabilidade a patógenos zoonóticos, especialmente devido à invasão de habitat e ao aumento das interações com humanos e outros animais silvestres.

## Introduction

Toxoplasmosis, caused by the protozoan *Toxoplasma gondii*, is a parasitic disease affecting a wide variety of warm-blooded mammals, including humans (Dubey & Desmonts, 1987; Dubey, 2008). Felids are definitive hosts that play an essential role in parasite dissemination by shedding oocysts into the environment (Dubey, 2022). Infection occurs through the ingestion of sporulated oocysts, the consumption of infected tissues, or transplacental transmission (Dubey, 2022). Wild animals in natural environments tend to be infected by oocysts shed by felids, either through contaminated water or raw meat. In contrast, managed care environments (e.g., bioparks, zoological parks) often facilitate rapid disease spread via contaminated water, raw food, or improper care practices (e.g., the use of shared utensils between enclosures) (Carme et al., 2009; Dubey, 2022). However, closer monitoring of zoos often enables timely disease detection (Casagrande et al., 2013).

Numerous studies have documented *T. gondii* infections in animals, including New World nonhuman primates (NWNHPs) (Dietz et al., 1997; Garcia et al., 2005; Casagrande et al., 2013; Amendoeira et al., 2022; Schiffler et al., 2023; Marian et al., 2024), marsupials (Canfield et al., 1990; Horta et al., 2018; Spriggs et al., 2020), wild felids (Basso et al., 2005; Villar-Echarte et al., 2021), rodents (Morales et al., 1996; Gennari et al., 2015; Horta et al., 2018), and domestic animals (Henker et al., 2022).

Moreover, high mortality rates have been observed in NWNHPs infected with *T. gondii* in animals under human care, with animals typically succumbing within hours of showing symptoms, such as weakness or lethargy (Dietz et al., 1997; Carme et al., 2009; Amendoeira et al., 2022). Sudden death is common in many cases, making postmortem examinations essential for diagnosis. Histopathological analyses often reveal *T. gondii* bradyzoites or tachyzoites in various tissues, with immunohistochemistry and molecular techniques (Richini-Pereira et al., 2016; Schiffler et al., 2023) used for confirmation.

*Coendou spinosus* (Rodentia: Erethizontidae; Paraguay hairy dwarf porcupines; Syn. *Sphiggurus villosus*) is a rodent species endemic to Southern Brazil. These arboreal animals live near forest edges, often close to human settlements, thus increasing their chances of encountering humans (Jorge et al., 2016). Such proximity results in frequent rescues from urban areas or backyards and roadkill incidents, with rescued animals usually sent to rehabilitation centers (Teixeira et al., 2013).

Herein, we report cases of fatal toxoplasmosis in *C. spinosus*, with a focus on an outbreak in a local zoo, and the pathological and genotypic findings for these cases.

## Material and Methods

### Animals sampled

Between September 2016 and October 2019, eight *C. spinosus* (CS) individuals were collected and analyzed at the Laboratório de Protozoologia e Rickettsioses Vetoriais, located at the Faculdade de Veterinária, Universidade Federal do Rio Grande do Sul (UFRGS) (Table 1). The animals were assigned the identification numbers CS1 to CS8. Specimens CS1, CS3, CS4, and CS5 were injured porcupines found in semi-rural areas and were referred to the Núcleo de Conservação e Reabilitação de Animais Silvestres (PRESERVAS-UFRGS). Individual CS2 was considered a roadkill case. Individuals CS6, CS7, and CS8 were post-mortem samples from a local zoo in the Zoológico Municipal de Cachoeira do Sul, Rio Grande do Sul, Brazil. In the Zoológico Municipal de Cachoeira do Sul, the animals underwent a rigorous clinical evaluation daily, conducted by the responsible veterinary physician, as part of the activities.

**Table 1:** Summary of clinical and laboratory findings in *Coendou spinosus* from the present study.

**Animal ID**	**Origin**	**Clinical signs**	**Histopathological findings**	***Toxoplasma gondii* detection (tissue samples)**	**Nested PCR (nPCR)**	**Genotyping**
**CS1**	Rehabilitation center	Not reported	**-**	-	Positive	Not performed
**CS2**	Roadkill	Not reported	**-**	-	Positive	Not performed
**CS3**	Rehabilitation center	Not reported	**-**	-	Positive	Not performed
**CS4**	Rehabilitation center	Not reported	**-**	-	Positive	Not performed
**CS5**	Rehabilitation center	Not reported	**-**	-	Positive	Not performed
**CS6**	Zoological park	Severe weakness, sudden death	CNS: Liquefactive necrosis and deposition of cellular debris, mild perivascular inflammatory infiltrate of lymphocytes, plasma cells, and macrophages Heart: mild necrosis, mild inflammatory infiltrate of lymphocytes and plasma cells. Kidneys: Moderate multifocal interstitial inflammatory infiltrate of lymphocytes and plasma cells.	Brain, heart, lung, liver, kidney (cysts and tachyzoites)	Positive	Not performed
**CS7**	Zoological park	Severe weakness, sudden death	CNS: Gliosis, inflammatory infiltrate composed of lymphocytes, plasma cells, and macrophages, and deposition of necrotic cellular debris. Mild perivascular inflammatory infiltrate of lymphocytes, plasma cells, and macrophages Heart: mild inflammatory infiltrate of lymphocytes and plasma cells	Brain, heart, lung, liver, kidney, adrenal (cysts and tachyzoites)	Positive	Not performed
**CS8**	Zoological park	Severe weakness	Gliosis, deposition of necrotic cellular debris, an mild perivascular inflammatory infiltrate of lymphocytes	Brain (cysts and tachyzoites)	Positive	Atypical genotype (RFLP)

### Histopathological and immunohistochemistry analyses

Necropsy examinations were performed at the Setor de Patologia Veterinária (UFRGS) using organ samples (brain, heart, liver, lung, and spleen) fixed in 10% formalin, embedded in paraffin, and sectioned for histopathological analysis. Immunohistochemistry was performed using anti-*T. gondii* antibodies (1:1,000; VMRD®, Pullman, WA, USA), employing the streptavidin-biotin peroxidase method with chromogen AEC (Biocare, Pacheco, CA, USA). Positive controls were included, and negative controls were incubated with phosphate-buffered saline instead of the primary antibodies.

### Molecular and genotyping assays

For molecular analysis, DNA was extracted from 10 mg of brain tissue from each animal, using an PureLink Genomic DNA Mini Kit (Invitrogen, Carlsbad, CA, USA). The DNA concentration was determined using a NanoDrop spectrophotometer (Thermo Fisher Scientific, Waltham, MA, USA), and nested polymerase chain reaction (nPCR) targeting the *T. gondii ITS-1* gene region was performed (Soares et al., 2011). Positive samples (CS8) underwent PCR-restriction fragment length polymorphism (RFLP) genotyping using the following 11 markers: 3′-*SAG2*, *SAG3*, *GRA6*, *C22-8*, *C29-2*, *L358*, *SAG1*, *BTUB*, *PK1,* alt. *SAG2* and APICO (Su et al., 2006). The genotypes were determined by comparing the amplified products with those of the positive control strains GTI (type I), PTG (type II), and CTG (type III) (Dardé et al., 1992). The standard strains were provided by researcher Chunlei Su from the UT College of Veterinary Medicine, Knoxville, United States. Samples were subjected to 2.5–3% agarose gel electrophoresis and visualized using an ultraviolet light transilluminator. The suggested genotype was compared to genotypes previously deposited in *ToxoDB* (Harb et al., 2020).

## Results

**Figure 1:**
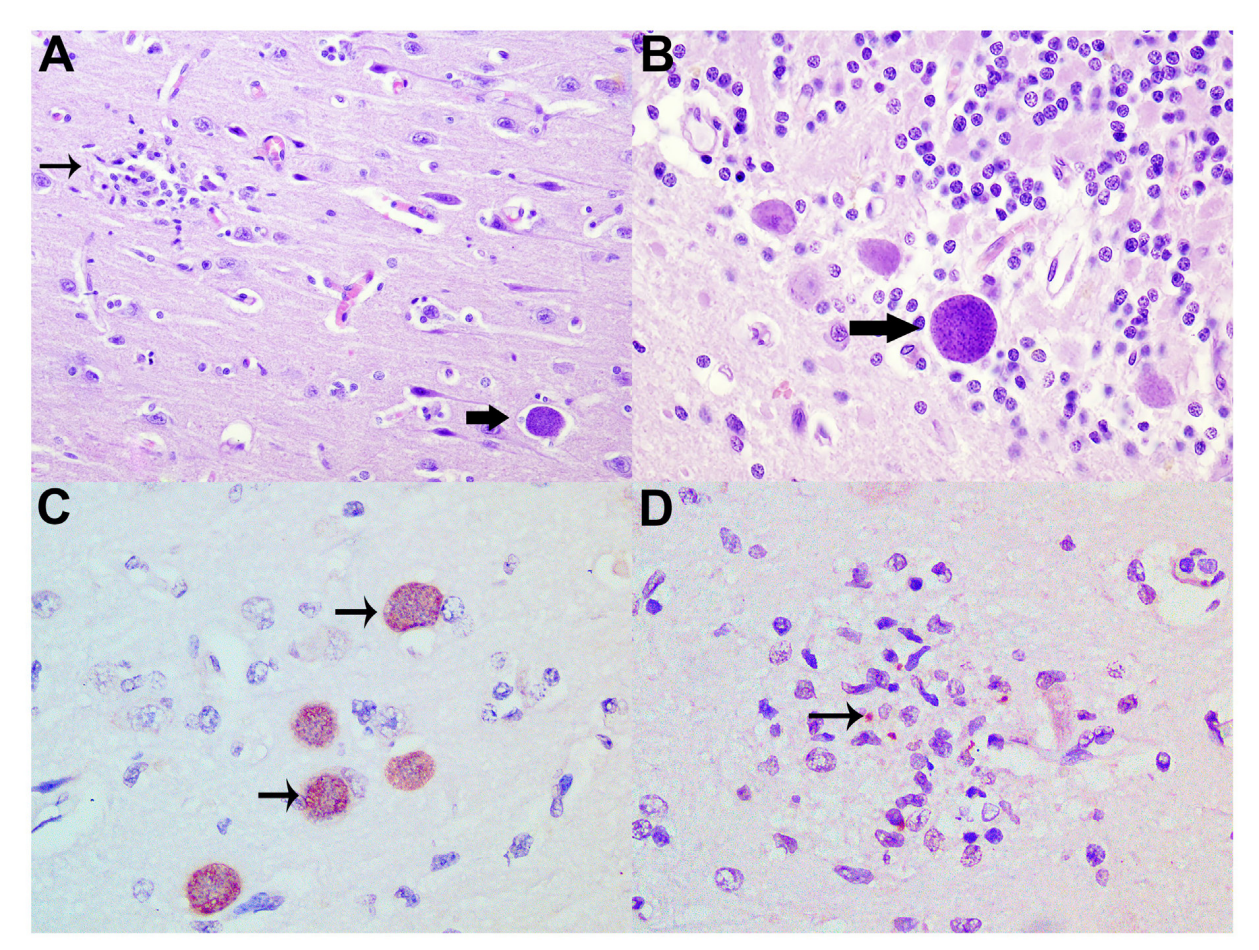
Toxoplasmosis in *Coendou spinosus*. Brain, histopathology and immunohistochemistry. **A.** Focal gliosis (thin arrow) and parasitic structures compatible with *Toxoplasma gondii* (thick arrow). H&E, 200×. **B.** Tissue cyst with a thick wall containing numerous elongated basophilic bradyzoites (arrow). H&E, 400×. **C.** Immunolabeling of *T. gondii* cysts (arrows). AEC chromogen, 400×. **D.** Immunolabeling of *T. gondii* tachyzoites (arrow). AEC chromogen, 600×.

The veterinarian responsible for the zoo noted that the clinical signs in *C. spinosus* range from severe weakness to sudden death, with necropsy revealing poor body condition. Histopathological examinations of all animals revealed extensive necrotic lesions in the telencephalon and cerebellum, accompanied by moderate gliosis (Figure 1A) and lymphoplasmacytic infiltrates, as well as mild perivascular cuffs with lymphocytes, plasma cells, and macrophages. Numerous parasitic structures morphologically consistent with *T. gondii* were intermixed with areas of necrosis and gliosis. These structures were represented by oval cysts (Figure 1B), measuring 25 to 40 µm in diameter, lined by a thick membrane, and they were filled with elongated basophilic structures (bradyzoites) (Table 1). Additionally, numerous elongated basophilic structures, measuring approximately 1 to 2 µm in length, morphologically consistent with *T. gondii* tachyzoites, were seen freely, especially in areas of necrosis and gliosis. In the heart, there was mild necrosis (CS6) and lymphocyte and plasma cell infiltration, along with a mild quantity of tachyzoites and numerous cysts (CS6 and CS7). Kidneys (CS6) there is moderate multifocal interstitial inflammatory infiltrate composed of lymphocytes and plasma cells. The lungs (CS6 and CS7), liver (CS6 and CS7), kidneys (CS6 and CS7), and adrenal glands (CS7) also exhibited *T. gondii* cysts (bradyzoites). Immunohistochemistry showed strong positive staining in cysts containing bradyzoites (Figure 1C) and tachyzoites (Figure 1D).

The nPCR confirmed the presence of *T. gondii* DNA in all positive cases, and subsequent genotyping revealed a previously undescribed atypical genotype profile detected in the animal CS8, underscoring the need for further genetic characterization. The genotypic characterizations determined using the RFLP method for the isolates were as follows: 3′-*SAG2* (type I or II), *SAG3* (type III), *GRA6* (type II), *C22-8* (type I), *C29-2* (type III), *L358* (type III), *SAG1* (type I), *BTUB* (type III), *PK1* (type III), alt. *SAG2* (type II), and *APICO* (type I or III). Although characterized as an atypical genotype, the markers *BTUB*, *PK1*, alt. *SAG2*, and *APICO* differed from those previously deposited in *ToxoDB*, and there was no evidence of clinical presentation in animals infected with this genotype (Table 2). Genotyping was performed only for the animal CS8, as it was not possible to evaluate the restriction patterns of other samples due to the low amount of DNA observed in electrophoresis.

**Table 2:** Genotypic profiles of *Toxoplasma gondii* identified in tissue samples from *Coendou spinosus* in the present study (sample CS8).

Animal ID	3 SAG2	SAG3	Gr6	C22-8	C 29-2	L358	SAG 1	BTUB	PK1	ALT SAG2	APICO	Reference
8	I or II	III	II	I	III	III	I	III	III	II	I or III	Su et al. (2006)

## Discussion

This case report documents the occurrence of clinical toxoplasmosis in *C. spinosus* in Brazil, marking a significant finding because of the severe outcomes observed. All five necropsied porcupines exhibited signs of severe debilitation, including general weakness and anorexia, and died immediately after symptom onset. The rapid disease progression in individuals CS6 and CS7, which experienced sudden death after nonspecific signs, aligns with previous reports on the acute progression of toxoplasmosis in NWNHP and Australian marsupials (Canfield, et al., 1990; Carme et al., 2009; Dietz et al., 1997).

A previous study by Santos et al. (2022) described *T. gondii* infection in *Sphiggurus spinosus* (syn. *Coendu spinosus*), with moderate parasite load and mild-to-moderate non-suppurative encephalitis. Cysts were restricted mainly to the brain and adrenal gland, and the infection was not fatal. In contrast, our cases in *C. spinosus* involved acute clinical deterioration and death, with widespread lesions in the brain and multiple peripheral organs. Genotypically, Santos et al. (2022) identified partial similarity to known ToxoDB genotypes (#6, #80, #126, #244), while our isolate presented a previously undescribed atypical profile with unique allele combinations at several loci. These findings suggest distinct strain characteristics and reinforce the importance of monitoring *T. gondii* diversity in wild porcupines. Additionally, a similar fatal case was described in *Coendou mexicanus* from a zoological park in Costa Rica, with disseminated lesions in the heart, lungs, liver, and kidneys and confirmation by immunohistochemistry (Morales et al., 1996). However, no genotypic data were provided.

The classification of *T. gondii* infections in NWNHPs, based on their response to the infection (Catão-Dias et al., 2013) includes Group I, which exhibits almost 100% mortality with little to no serological response and mainly observed in the family Callitrichinae (*Saguinus*, *Leontopithecus*, *Callithrix*), as well as Group II (e.g., *Alouatta*) showing partial survival, and Group III (e.g., *Cebus*) that shows mild disease (Catão-Dias et al., 2013; Paula et al., 2020).A similar pattern was observed in the present study. Although we report a rodent species, the pathological findings—characterized by acute disease progression, fatal outcome, and extensive CNS lesions with abundant parasitic forms in all five animals—closely resemble the Group I profile described in highly susceptible NWNHPs. In addition, these findings are consistent with those of previous studies that documented fatal toxoplasmosis with parasitic structures in various organs of NWNHPs, such as the lungs and liver (Epiphanio et al., 2003; Paula et al., 2020), which similar lesions were found in *C. spinosus* CS6 and CS7.

Similarly, at the same local zoo where this outbreak occurred, brown howler monkeys (*Alouatta guariba clamitans*) exhibited typical responses to toxoplasmosis, characterized by rapid mortality following symptom onset and more pronounced serological responses. Indeed, toxoplasmosis in NWNHPs often results in high-mortality outbreaks; for example, seven *Alouatta* spp. were reported to have died over a 34-day period, with deaths occurring 2–15 days apart (Santana et al., 2021). This mortality pattern suggests possible direct contact transmission, especially when animals share enclosures. In another outbreak, 25 squirrel monkeys (*Saimiri sciureus*) died within 48 hours of showing clinical signs, including asthenia and respiratory distress (Carme et al., 2009). Same pattern was observed with a highly virulent *T. gondii* strain (i.e., TgBgHmBrRJ1) isolated from a captive black-and-gold howler monkey (*Alouatta caraya*) at the Primatology Center of Rio de Janeiro State, Southeastern Brazil (Amendoeira et al., 2022).

Furthermore, rodents act as intermediate and reservoir hosts for *T. gondii* as they maintain chronic infections and are the primary sources of infection in felid hosts (Rabiee et al., 2018). Infection with *T. gondii* in rodents often results in behavioral changes that facilitate the transmission cycle (Gotteland et al., 2014). However, the role of wild rodents under human care remains unknown, especially from a public health perspective.

The high susceptibility of arboreal animals to toxoplasmosis may be linked to their habitat and behavior. Porcupines, which spend a considerable amount of time in trees, are likely to face increased exposure to contaminated food and water when descending to the ground (Dubey, 1998). Even in managed care settings, where the risks should theoretically be reduced through proper enclosure maintenance, of a Geoffroyi’s cat (*Leopardus geoffroyi*) and handlers at local zoos may potentially contribute to environmental contamination. Furthermore, untreated water and raw vegetables (e.g., leafy greens), can act as important vehicles for the transmission of *T. gondii* oocysts when not adequately washed or sourced from safe and controlled environments, and their role in outbreaks should not be underestimated (Dubey, 2021).

Toxoplasmosis outbreaks in zoos have been documented globally. A serosurvey conducted across eight zoos in Spain between 2007 and 2019 found that 42% of the 393 animals tested were seropositive for *T. gondii*, with carnivorous species at a higher risk because of their feeding habits (Cano-Terriza et al., 2020). In a longitudinal analysis of 39 animals, 46.2% remained consistently seropositive, with five animals seroconverting during the study period. This highlights the widespread circulation of *T. gondii* in zoo settings, posing a risk to animals, personnel, veterinarians, and visitors. In contrast, studies of wild and captive marsupials and wallaroos have shown high susceptibility to toxoplasmosis, despite differing ecological contexts (Boorman, et al., 1977; Canfield, et al., 1990).

Genotyping of the *T. gondii* isolates from *C. spinosus* allowed the identification of an atypical genotype in a zoo. This is the first report of genotypic characterization of this protozoan in Southern Brazil and the findings highlight the need for prophylactic measures against *T. gondii* infection in *C. spinosus*, particularly in zoos.

Given the prevalence of toxoplasmosis outbreaks in zoos, primary control measures should focus on isolating felids to prevent contamination through feces containing *T. gondii* oocysts, which may spread into other enclosures, especially those housing NWNPs and rodents (Dubey, 2022). Additional precautions should include daily removal of feline feces to prevent oocyst sporulation, ensuring that handlers wear protective clothes, and avoiding cross-contamination by using footwear in different enclosures.

Furthermore, this study reports fatal cases of toxoplasmosis in *C. spinosus* and provides the first genotypic characterization of *T. gondii* in this species in Southern Brazil. The findings reveal an acute clinical course with high mortality, widespread tissue lesions, and an atypical genotype not previously recorded in ToxoDB. These results highlight the susceptibility of *C. spinosus* to *T. gondii* infection and highlight the importance of surveillance in wildlife under human care. The identification of a virulent, atypical strain in a zoo park setting also reinforces the need for stricter biosecurity protocols, including the monitoring of food, water, and environmental hygiene. Considering the zoonotic potential of *T. gondii*, enhanced preventive measures are essential to protect both animal and public health.

## Data Availability

The authors inform that all data are available in the text

## References

[B001] Amendoeira MRR, Arruda IF, Moreira SB, Ubiali DG, Barbosa ADS, Pena HFJ (2022). Isolation and genetic characterization of *Toxoplasma gondii* from a captive black-and-gold howler monkey (*Alouatta caraya* Humboldt, 1812) in Brazil. Int J Parasitol Parasites Wildl.

[B002] Basso W, Edelhofer R, Zenker W, Möstl K, Kübber-Heiss A, Prosl H (2005). Toxoplasmosis in Pallas’ cats (*Otocolobus manul*) raised in captivity. Parasitology.

[B003] Boorman GA, Kollias GV, Taylor RF (1977). An outbreak of toxoplasmosis in wallaroos (*Macropus robustus*) in a California zoo. J Wildl Dis.

[B004] Canfield PJ, Hartley WJ, Dubey JP (1990). Lesions of toxoplasmosis in Australian marsupials. J Comp Pathol.

[B005] Cano-Terriza D, Almería S, Caballero-Gómez J, Jiménez-Martín D, Castro-Scholten S, Dubey JP (2020). Exposure to *Toxoplasma gondii* in zoo animals in Spain. Prev Vet Med.

[B006] Carme B, Ajzenberg D, Demar M, Simon S, Dardé ML, Maubert B (2009). Outbreaks of toxoplasmosis in a captive breeding colony of squirrel monkeys. Vet Parasitol.

[B007] Casagrande RA, Silva CE, Pescador CA, Borelli V, Souza JC, Souza ER (2013). Toxoplasmose em primatas neotropicais: estudo retrospectivo de sete casos. Pesq Vet Bras.

[B008] Catão-Dias JL, Epiphanio S, Kierulff MCM, Brinkworth J, Pechenkina K (2013). Primates, pathogens, and evolution. developments in primatology: progress and prospects..

[B009] Dardé ML, Bouteille B, Pestre-Alexandre M, Darde ML (1992). Isoenzyme analysis of 35 *Toxoplasma gondii* isolates and the biological and epidemiological implications. J Parasitol.

[B010] Dietz HH, Henriksen P, Bille-Hansen V, Henriksen SA (1997). Toxoplasmosis in a colony of New World monkeys. Vet Parasitol.

[B011] Dubey JP, Desmonts G (1987). Serological responses of equids fed *Toxoplasma gondii* oocysts. Equine Vet J.

[B012] Dubey JP (1998). Advances in the life cycle of *Toxoplasma gondii.*. Int J Parasitol.

[B013] Dubey JP (2008). The history of *Toxoplasma gondii*: the first 100 years. J Eukaryot Microbiol.

[B014] Dubey JP (2021). Toxoplasmosis of animals and humans..

[B015] Dubey JP (2022). Clinical toxoplasmosis in zoo animals and its management. Emerg Anim Spe.

[B016] Epiphanio S, Sinhorini IL, Catão-Dias JL (2003). Pathology of toxoplasmosis in captive new world primates. J Comp Pathol.

[B017] Garcia JL, Svoboda WK, Chryssafidis AL, De Souza Malanski L, Shiozawa MM, De Moraes Aguiar L (2005). Sero-epidemiological survey for toxoplasmosis in wild New World monkeys (*Cebus* spp.; *Alouatta caraya*) at the Paraná river basin, Paraná State, Brazil. Vet Parasitol.

[B018] Gennari SM, Ogrzewalska MH, Soares HS, Saraiva DG, Pinter A, Nieri-Bastos FA (2015). *Toxoplasma gondii* antibodies in wild rodents and marsupials from the Atlantic Forest, state of São Paulo, Brazil. Rev Bras Parasitol Vet.

[B019] Gotteland C, Chaval Y, Villena I, Galan M, Geers R, Aubert D (2014). Species or local environment, what determines the infection of rodents by *Toxoplasma gondii?*. Parasitology.

[B020] Harb OS, Kissinger JC, Roos DS, Weiss LM, Kim K (2020). Toxoplasma gondii: the model apicomplexan – perspectives and methods..

[B021] Henker LC, Vogel FSF, Cecco BS, Santos IR, Roman IJ, Fernandes FD (2022). Abortion outbreak in a sheep flock caused by *Toxoplasma gondii* clonal type III. Parasitol Res.

[B022] Horta MC, Guimarães MF, Arraes-Santos AI, Araujo AC, Dubey JP, Labruna MB (2018). Detection of anti-*Toxoplasma gondii* antibodies in small wild mammals from preserved and non-preserved areas in the Caatinga biome, a semi-arid region of Northeast Brazil. Vet Parasitol Reg Stud Rep.

[B023] Jorge LMA, Bernardes F, Lamy F, Balassiano LKA, Towersey L, Hay R (2016). Clinical manifestation, histopathology, and imaging of traumatic injuries caused by Brazilian porcupine (*Sphiggurus villosus*) quills. Case Rep Dermatol Med.

[B024] Marian L, Withoeft JA, Fornara MA, Pandolfo GW, Fernandes FD, Vogel FSF (2024). Toxoplasmosis outbreak caused by north American genotypes in captive black-tufted marmosets in southern Brazil. Vet Parasitol Reg Stud Rep.

[B025] Morales JA, Peña MA, Dubey JP (1996). Disseminated toxoplasmosis in a captive porcupine (*Coendou mexicanus*) from Costa Rica. J Parasitol.

[B026] Paula NFD, Dutra KS, Oliveira AR, Santos DO, Rocha CEV, Vitor RWA (2020). Host range and susceptibility to *Toxoplasma gondii* infection in captive neotropical and Old‐world primates. J Med Primatol.

[B027] Rabiee MH, Mahmoudi A, Siahsarvie R, Kryštufek B, Mostafavi E (2018). Rodent-borne diseases and their public health importance in Iran. PLoS Negl Trop Dis.

[B028] Richini-Pereira VB, Marson PM, Silva RC, Langoni H (2016). Genotyping of *Toxoplasma gondii* and *Sarcocystis* spp. in road-killed wild mammals from the central western region of the state of São Paulo, Brazil. Rev Soc Bras Med Trop.

[B029] Santana CH, de Oliveira AR, Dos Santos DO, Pimentel SP, de Souza LD, Moreira LG (2021). Genotyping of *Toxoplasma gondii* in a lethal toxoplasmosis outbreak affecting captive howler monkeys (*Alouatta* sp.). J Med Primatol.

[B030] Santos ALM, Navas‐Suárez PE, Guerra JM, Ervedosa TB, Rivas L, Joppert A (2022). Toxoplasmosis in a free‐ranging hairy dwarf porcupine (*Sphiggurus spinosus*) with a potential novel genotype. Transbound Emerg Dis.

[B031] Schiffler FB, Pereira AHB, Moreira SB, Arruda IF, Moreira FRR, D’arc M (2023). Lessons from a multilaboratorial task force for diagnosis of a fatal toxoplasmosis outbreak in captive primates in Brazil. Microorganisms.

[B032] Soares RM, Lopes EG, Keid LB, Sercundes MK, Martins J, Richtzenhain LJ (2011). Identification of *Hammondia heydorni* oocysts by a heminested-PCR (hnPCR-AP10) based on the *H. heydorni* RAPD fragment AP10. Vet Parasitol.

[B033] Spriggs M, Jiang T, Gerhold R, Stedman N, López-Orozco N, Su C (2020). Genotype identification of *Toxoplasma gondii* in macropods from a zoological park in Florida, USA. J Zoo Wildl Med.

[B034] Su C, Zhang X, Dubey JP (2006). Genotyping of *Toxoplasma gondii* by multilocus PCR-RFLP markers: a high resolution and simple method for identification of parasites. Int J Parasitol.

[B035] Teixeira FZ, Printes RC, Fagundes JCG, Alonso AC, Kindel A (2013). Canopy bridges as road overpasses for wildlife in urban fragmented landscapes. Biota Neotrop.

[B036] Villar-Echarte G, Arruda IF, Barbosa AS, Guzmán RG, Augusto AM, Troccoli F (2021). *Toxoplasma gondii* among captive wild mammals in zoos in Brazil and Cuba: seroprevalence and associated risk factors. Rev Bras Parasitol Vet.

